# Effect of ethylene glycol dimethacrylate on swelling and on metformin hydrochloride release behavior of chemically crosslinked pH–sensitive acrylic acid–polyvinyl alcohol hydrogel

**DOI:** 10.1186/s40199-015-0123-8

**Published:** 2015-08-19

**Authors:** Muhammad Faheem Akhtar, Nazar Muhammad Ranjha, Muhammad Hanif

**Affiliations:** Faculty of Pharmacy, Bahauddin Zakariya University, P.O.Box: 60800, Multan, Pakistan

**Keywords:** Acrylic acid–polyvinyl alcohol hydrogel, Ethylene glycol dimethacrylate, Glutaraldehyde, Metformin hydrochloride, Dynamic swelling, Drug release

## Abstract

**Background:**

The present work objective was to prepare and to observe the effect of ethylene glycol dimethacrylate on swelling and on drug release behavior of pH-sensitive acrylic acid–polyvinyl alcohol hydrogel.

**Methods:**

In the present work, pH sensitive acrylic acid–polyvinyl alcohol hydrogels have been prepared by free radical polymerization technique in the presence of benzoyl peroxide as an initiator. Different crosslinker contents were used to observe its effect on swelling and on drug release. Dynamic and equilibrium swelling studies of prepared hydrogels were investigated in USP phosphate buffer solutions of pH 1.2, 5.5, 6.5 and 7.5 with constant ionic strengths. Hydrogels were evaluated for polymer volume fraction, solvent interaction parameter, molecular weight between crosslinks, number of links per polymer chain, diffusion coefficient, sol–gel fraction and porosity. To demonstrate the release pattern of the drug, zero-order, first-order, higuchi and korsmeyer-peppas models were applied. Quality and consistency of hydrogels was examined by FTIR and surface morphology of hydrogels was examined by SEM.

**Results:**

Decrease in swelling and in drug release was seen by increasing content of ethylene glycol dimethacrylate. A remarkable high swelling was observed at high pH indicating the potential of this hydrogel for delivery of drugs to intestine. By increasing the concentration of ethylene glycol dimethacrylate, porosity decreased. Order of release was observed first order in all cases and the mechanism was non–fickian diffusion. FTIR confirmed the formation of network. SEM results showed the incorporation of drug.

**Conclusion:**

The prepared hydrogels can be suitably used for targeted drug delivery to the intestine.

## Background

Hydrogel, three–dimensional crosslinked polymeric network, can swell and collapse reversibly in response to variables such as ionic strength, pH, electric field and temperature [1]. Hydrogels can be used as controlled release systems when they are in contact with any surface. This can happen through spaces inside the network and the matrix dissolution/disintegration effect [2].

Polyvinyl alcohol (PVA) is being extensively used in fields, such as: pharmaceutical (for the wound dressing systems); biomedical (as a scaffold supporting material for tissue engineering applications) and environmental (for the production of films for removal of heavy metal ions from water). Other applications comprise fuel cells, electrochemistry and agriculture. The –OH group on every second carbon atom on PVA backbone allows it to take part in many chemical crosslinking reactions, to interact with many other polymers by hydrogen bonding, and to form a hydrogel by the freeze thaw process. Excellent biocompatibility, noncarcinogenicity, biodegradability and non-toxicity are supplementary attractive properties of PVA. PVA is also useful in the pharmaceutical industries, where it is being used as a polymer for the loading/encapsulation and the subsequent release of cells, enzymes, proteins and a range of drugs [3].

Acrylic acid (AA) is a superabsorbent and a common pH-sensitive electrolyte. Because gels can be prepared at varying concentrations, AA based materials present huge potential for biomedical applications. They may be easily converted to a broad range of shapes and sizes. Prior to gel formation, other materials may be included into AA. AA polymers exhibit high tolerance in living cells. In addition, a glycoprotein i.e. mucin secreted locally that coats the mucosal surfaces forms hydrogen bonds with carboxylic groups of AA. AA is a fine applicant for many drug delivery routes e.g. nasal, ocular and oral due to its bioadhesive property. As, carboxylic groups of AA intermingle with different groups, attachment sites are created for a variety of therapeutics [4].

In the synthesis of a large number of hydrogels, crosslinkers are used, which interconnect the lineal polymeric chains establishing a three-dimensional network of chemical bonds among them. It is necessary that the polymer has certain groups in its structure that can be used as anchor points in order to form the network. The choice of crosslinker depends on the selected monomers, must have at least two reactive groups in its structure, in order to be able to crosslink different polymeric chains, normally tetrafunctional and hexafunctional compounds, such as ethylene glycol dimethacrylate (EGDMA) and 1,1,1,trimethylolpropane trimethacrylate, although other crosslinking agents have also been used such as ethylenediaminetetraacetic dianhydride and pentaerythritol triacrylate [5]. Glutaraldehyde (GA) has been extensively used for crosslinking polymers containing hydroxyl groups [6]. Hydrogels containing ionic network structure show pH–dependent swelling behavior [7].

In the present work, ethylene glycol dimethacrylate and glutaraldehyde crosslinked pH***–***sensitive acrylic acid–polyvinyl alcohol hydrogels were synthesized for drug delivery to intestine. Different quantities of crosslinking agent were used in order to evaluate its effects on swelling and on drug release.

## Methods

### Materials

Polymer used was polyvinyl alcohol (Mol.wt. 72000; degree of hydrolysis ≥98 %; Merck, Germany). The monomer used was acrylic acid (Sigma-Aldrich, Netherland). Crosslinkers used were ethylene glycol dimethacrylate (Sigma-Aldrich, Germany) and glutaraldehyde (Scharlau, Spain). Benzoyl peroxide (Fisher Scientific, UK) was used as an initiator and HCl (Fluka, Switzerland) was used as a catalyst. Distilled water was used as a solvent. Potassium dihydrogen phosphate was purchased from Merck, Germany. Metformin hydrochloride was gifted by Popular International (PVT) LTD., Karachi, Pakistan.

### Synthesis of pH sensitive AA–PVA hydrogels

**Fig. 1:**
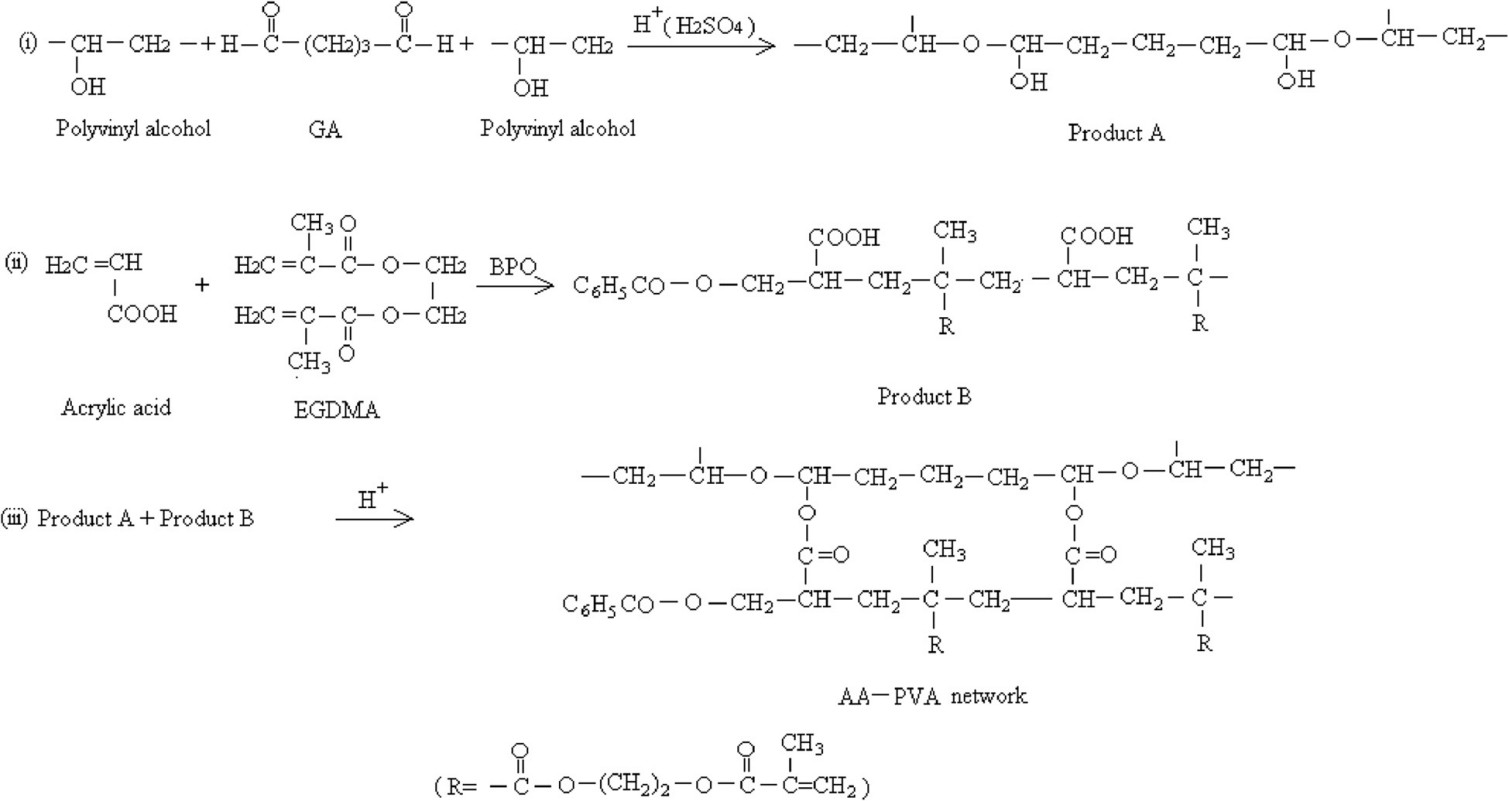
Possible structure of synthesized acrylic acid–polyvinyl alcohol hydrogel

AA–PVA hydrogels were prepared by the free radical polymerization technique. The process of hydrogel preparation was analogous to that reported [8] but with some essential modifications due to the properties of ingredients. A 10 % w/v PVA solution was prepared at 80 °C using reflux condenser. Mixing was continued until heated solution cooled to room temperature and then HCl and GA were added with continuous stirring at slow speed. This solution was named as solution A. Benzoyl peroxide was dissolved in acrylic acid and then varying amounts of EGDMA were added to this solution. After stirring, this solution was named as solution B. Both solutions were mixed very slowly to prevent the formation of air bubbles [9] and distilled water was added to make the final weight of the solution 100 g. Immediately after mixing the solution [9], the mixture was poured into Pyrex glass tubes having 150 mm length and 16 mm internal diameter to start polymerization. Nitrogen bubbling was done for 10–15 min to prevent obstruction in normal polymerization process by oxygen [10]. Glass tubes after being capped were placed in the water bath at a temperature regime of 45 °C for 1 h, 50 °C for 2 h, 55 °C for 3 h, 60 °C for 4 h and 65 °C for 5 h. To avoid auto-acceleration and air bubbles formation, there was a gradual increase in temperature from 45 °C to 65 °C. Then the tubes were cooled down and cylindrical hydrogels were removed from the tubes. 7 mm length disks were cut from each cylinder. Extensive washing of these discs with freshly distilled water was done for the unreacted material removal. Drying of disks was done at room temperature and then in an oven to constant weight at 45 °C and stored in a desiccator for further use. Figure 1 is showing the possible chemical structure of synthesized acrylic acid–polyvinyl alcohol hydrogel. A list of different formulations of AA–PVA hydrogel is given in Table 1.

**Table 1:** A list of different formulations of AA–PVA hydrogel

Sample code	AA	PVA	AA/PVA	EGDMA	GA
	(g/100 g solution)	(g/100 g solution)	(wt.%)	(g/100 g solution)	(g/100 g solution)
S_1_	21	7.38	74/26	0.082	0.01
S_2_	21	7.38	74/26	0.123	0.01
S_3_	21	7.38	74/26	0.165	0.01

### Buffer solutions preparation

pH 1.2, 5.5, 6.5 and 7.5 USP phosphate buffer solutions were prepared with potassium dihydrogen phosphate. 0.2 M HCl or NaOH solution was used to adjust the pH of these solutions. NaCl was used to keep the ionic strength of all the buffer solutions constant.

### Dynamic and equilibrium swelling study

Dynamic and equilibrium swelling study was done in 100 ml pH 1.2, 5.5, 6.5 and 7.5 buffer solutions. Dried and weighed hydrogel was kept in a desired pH solution. For dynamic swelling study, the swollen gel was taken out of the buffer solution, blotted with tissue paper, weighed and then placed back in the same buffer solution, at regular interval upto 8 h. Following equation was used to calculate the swelling ratio [11]:

1 $$ \mathrm{q}={\mathrm{W}}_{\mathrm{t}}/{\mathrm{W}}_{\mathrm{d}} $$

W_t_ is the swollen gel weight at time t and W_d_ is the dry gel initial weight. For equilibrium swelling, the swollen gels were weighed daily until they reach constant weight which took almost 2 weeks.

### Network parameters of AA–PVA hydrogels

#### Polymer volume fraction

Polymer volume fraction (*v*_2,s_) is the fluid amount that a hydrogel can absorb in the equilibrium swollen state. Following equation was used to calculate *v*_2,s_ [12]:

2 $$ {v}_{2,s}=\left[\frac{vp}{vgel}\right] $$

*v*_*p*_ is the dried hydrogel volume and *v*_*gel*_ is the volume in swollen state.

#### Solvent interaction parameter (χ)

Following equation was used to calculate *χ* values [13]:

3 $$ \chi =\frac{1}{2}+\frac{v}{2,s 3} $$

#### Molecular weight between crosslinks (M_c_)

*M*_*c*_ was calculated by the following equation [12]:

4 $$ M\;_c=\frac{Mr}{2X} $$

where *M*_*r*_ is the polymer repeating unit molar mass and *X* is the degree of crosslinking.

*M*_*r*_ was calculated by the following equation [14]:

5 $$ {M}_r=\frac{n_{PVA}{M}_{PVA}+{n}_{AA}{M}_{AA}}{n_{PVA}+{n}_{AA}} $$

where *n*_*PVA*_ and *n*_*AA*_ are the number of moles of PVA and AA, respectively while *M*_*PVA*_ and *M*_*AA*_ are PVA and AA molar masses, respectively.

#### Number of links per polymer chain (N)

The equation used is given below [15]:

6 $$ N=\frac{2{M}_c}{M_r} $$

#### Diffusion coefficient

Usually, diffusion is the mechanism of drug release from hydrogels. Equation used to calculate water diffusion coefficient is given below [16]:

7 $$ D=\pi {\left(\frac{h.\theta }{4.{Q}_{eq}}\right)}^2 $$

where *D* is the hydrogel diffusion coefficient, *θ* is the swelling curve linear part slope, *Q*_eq_ is the equilibrium swelling ratio and *h* is the sample thickness before swelling.

#### Sol–gel fraction

To remove uncrosslinked polymer, hydrogel unwashed samples were cut into 3–4 mm diameter pieces, dried in an oven at 45 °C to constant weight (*W*_*o*_), and subjected to soxhlet extraction with deionized water for 4 h. Extracted gels were dried again at 45 °C in an oven to constant weight (*W*_*1*_). Following equations were used to calculate gel fraction [17]:

8 $$ \mathrm{Sol}\ \mathrm{fraction}\left(\%\right)=\left[\frac{W_o-{W}_1}{W_o}\right]X 100 $$

9 $$ \mathrm{Gel}\kern0.5em \mathrm{fraction}\left(\%\right)=100\hbox{--} \mathrm{sol}\kern0.5em \mathrm{fraction} $$

#### Porosity measurement

Solvent replacement method was used for porosity measurement. Dried and weighed hydrogel was soaked in absolute ethanol over night and weighed after blotting excess ethanol from the surface and the porosity was calculated by the following equation [18]:

10 $$ Porosity=\frac{\left({M}_2-{M}_1\right)}{\rho V}\times 100 $$

*M*_*1*_ and *M*_*2*_ are the hydrogel masses before and after immersion in ethanol, respectively: *ρ* is the absolute ethanol density and *V* is the hydrogel final volume.

### Loading of metformin hydrochloride into crosslinked AA–PVA hydrogels

Weighed and dried hydrogel samples were placed in 1 % w/v solution of metformin hydrochloride. Metformin hydrochloride solution was prepared by dissolving the drug in USP phosphate buffer solution of pH 7.5. After attaining the equilibrium swelling, hydrogel samples were dried first at room temperature and then in an oven at 45 °C to constant weight.

### Determination of metformin hydrochloride loading

Three methods were applied. Following equation was used to determine drug loading by the first method:

11 $$ \mathrm{Amount}\ \mathrm{of}\ \mathrm{drug}={\mathrm{W}}_{\mathrm{D}}\hbox{--} {\mathrm{W}}_{\mathrm{d}} $$

Weights of dried hydrogels before and after immersion in drug solution are W_d_ and W_D_, respectively. In the second method, drug entrapped was calculated by repeatedly extracting the weighed quantity of loaded gels using USP phosphate buffer solution (pH 7.5). Each time fresh 50 ml USP phosphate buffer solution (pH 7.5) was used until drug exhaustion. Drug concentration was determined spectrophotometrically. Drug present in all portions of the extracts was considered as the drug amount loaded. Weighed gel disk was placed in drug solution up to equilibrium swelling, in the third method. Loaded gel was weighed again after blotting with filter paper. Difference in weight before and after swelling is the weight of drug solution. Dividing the weight of drug solution with the density of drug solution gave us the volume of drug solution. So, amount of drug was easily calculated from the volume of drug solution [4].

### Metformin release studies

The weighed hydrogel disks were immersed separately in 500 ml 0.05 M USP phosphate buffer solutions of pH 1.2, 5.5 and 7.5 at 37 °C and dissolution medium was stirred at a rate of 100 rpm for maintaining a uniform drug concentration (Dissolution apparatus, Pharmatest; PT–Dt 7, Germany). Metformin HCl release study was conducted at 218 nm up to 12 h (UV–VIS spectrophotometer, IRMECO, UV–VIS U2020) [4].

### Analysis of drug release pattern

For the analysis of release of metformin hydrochloride, zero-order [19], first-order [20], higuchi [21] and korsmeyer-peppas [22] models were applied. To get an insight into the solute release mechanism, the release profile was analyzed using the peppas semi-empirical power equation [22]. Following equations were used for release calculations.

12 $$ \mathrm{Zero}\hbox{-} \mathrm{order}\kern0.5em \mathrm{kinetics}:\kern1em {\mathrm{F}}_{\mathrm{t}}={\mathrm{K}}_{\mathrm{o}}\mathrm{t} $$

where F represents the fraction of drug release in time t and K_o_ is the zero-order release constant.

13 $$ \mathrm{F}\mathrm{irst}\hbox{-} \mathrm{order}\kern0.5em \mathrm{kinetics}:\kern1em  \ln \left(1\hbox{-} \mathrm{F}\right)=\hbox{-} {\mathrm{K}}_1\mathrm{t} $$

where F represents the fraction of drug release in time t and K_1_ is the first-order release constant.

14 $$ \mathrm{Higuchi}\kern0.5em \mathrm{model}:\kern1em \mathrm{F}={\mathrm{K}}_2{\mathrm{t}}^{\frac{1}{2}} $$

where F represents the fraction of drug release in time t and K_2_ is the higuchi constant.

15 $$ \mathrm{Korsmeyer}\hbox{-} \mathrm{peppas}\kern0.5em \mathrm{model}:\kern1.25em {\mathrm{M}}_{\mathrm{t}}/\mathrm{M}\propto ={\mathrm{K}}_3{\mathrm{t}}^{\mathrm{n}} $$

M_t_ is the mass of water absorbed at any time t; M∝ is the amount of fluid intake at equilibrium; K_3_ is the kinetic constant and n is the swelling exponent.

### FTIR spectroscopic analysis

The crushed hydrogel samples were mixed with potassium bromide (Merck IR spectroscopy grade) in 1:100 proportions and dried at 45 °C. The mixtures were compressed to a 12 mm semitransparent disk by a pressure of 65 kN (Pressure gauge, Shimadzu) for 1 min. The FTIR spectra were recorded over the wavelength range 4,000–400 cm^−1^ using FTIR spectrometer (FTIR 8400 S, Shimadzu).

### Scanning electron microscopy (SEM)

The morphology of AA–PVA hydrogel and drug loaded AA–PVA hydrogel was observed using scanning electron microscope JSM–6480.

## Results and discussion

### pH impact on swelling and on drug release behavior of AA–PVA hydrogels

The polymer chains absorb water in the presence of an aqueous solution, and the association/dissociation of various ions to polymer chains cause the IPN (Interpenetrating polymeric network) to swell. Ionic hydrogels are those hydrogels which have ionizable functional groups. Anionic gels swell at basic pH values and collapse at acidic pH values. The pKa of AA is 4.26. Carboxylic groups of the network ionize and attract cations to replace the H^+^ ions, as the pH of the environmental solution is above its pKa. This successfully increases the concentration of free ions inside the gel. So, the ionic swelling pressure will increase and so does the swelling. Additionally, the carboxylate anions cause more hydrophilicity and electrostatic repulsion to the polymer segments in the hydrogel. So, by the increase in pH, the AA–PVA hydrogels swelled speedily due to more swelling driving force caused by the electrostatic repulsion between the ionized carboxylate groups [23–25]. Manifestation of the swelling ratios (dynamic and equilibrium) of AA–PVA hydrogels is given in Table 2.

**Table 2:** Swelling coefficients (Dynamic and equilibrium) of AA–PVA hydrogels using EGDMA and GA as crosslinkers

Sample codes	Dynamic swelling coefficients				Equilibrium swelling coefficients			
	1.2 pH	5.5 pH	6.5 pH	7.5 pH	1.2 pH	5.5 pH	6.5 pH	7.5 pH
S_1_	2.9	3.29	4.32	4.82	10.19	11.72	18.11	23.85
S_2_	2.58	2.9	4.08	4.33	10.07	11.53	17.67	21.4
S_3_	2.26	2.56	3.76	4	9.88	11.25	17.03	19.8

Table 3 is showing metformin amount loaded in various samples. To see the pH effect on drug release behavior, dissolution profiles were obtained in buffer solutions (pH 1.2, 5.5 and 7.5). Drug release increased as the medium pH increased, in all samples. Table 4 is showing the effect of pH on dug release after 12 h drug release study. Drug release can be correlated with the AA–PVA hydrogel samples swelling behavior where the swelling increased when the medium pH increased.

**Table 3:** Metformin amount loaded in different samples of AA–PVA hydrogel

Sample code	Amount of metformin loaded		
	(g/g of dry gel)		
	Swelling method	Extraction method	Weight method
S_1_	0.2194	0.22	0.2157
S_2_	0.2065	0.2093	0.2029
S_3_	0.1999	0.1995	0.1921

**Table 4:** Effect of pH on dug release after 12 h drug release study

Sample	Time (hours)	pH 1.2	pH 5.5	pH 7.5
S_1_	12	26.02 %	49.77 %	80.42 %
S_2_	12	25.48 %	47.48 %	75.95 %
S_3_	12	22.81 %	45.27 %	71.76 %

### Effect of EGDMA content on swelling and on drug release behavior of AA–PVA hydrogel

**Fig. 2:**
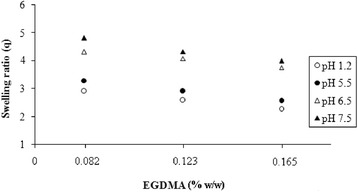
Swelling behavior after 8 h of AA–PVA hydrogel with different EGDMA content

The swelling of three hydrogel samples (S_1_–S_3_) was studied at different pH values as a function of different feed EGDMA concentrations. Fig. 2 is showing EGDMA content effect on the dynamic swelling coefficient keeping PVA and AA contents constant. A clear picture can be seen that the swelling ratio decreased by increasing the amount of EGDMA. This was due to the fact that as the crosslinker content increased, there was a decrease in the network mesh size and an increase in the stability of the network resulting in lower swelling [26, 27].

**Fig. 3:**
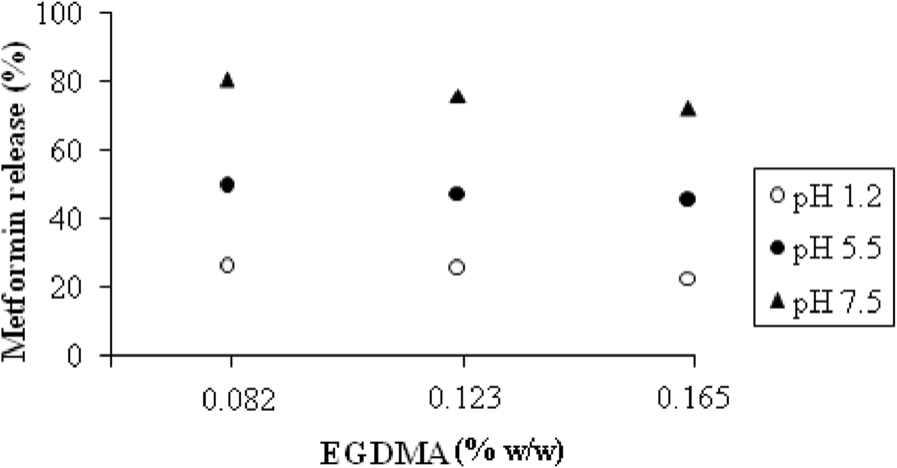
Effect of EGDMA content on metformin HCl release after 12 h from AA–PVA hydrogel

By increasing EGDMA concentration, a decrease in drug release at all pH values was observed. It can be correlated with the swelling behavior. The effect of EGDMA content on drug release is shown in Fig. 3.

### Network parameters of AA–PVA hydrogels

The key parameters to characterize crosslinked swollen network are *M*_*C*_ and *v*_2,s_ because *M*_*C*_ is a gauge of degree of crosslinking of the polymer while *v*_2,s_ evaluates the liquid amount retained by the network. During mathematical modeling, equilibrium swelling data of pH 7.5 was used. Values of *v*_2,s_, χ, *M*_*C*_, *M*_*r*_, *N* and *D* are elaborated in Table 5.

**Table 5:** Network parameters of AA–PVA hydrogels

Sample code	*v*_*2,s*_	Χ	*M*_*C*_	*M*_*r*_	*N*	*D* (cm^2^/sec.)	Gel fraction (%)	Porosity (%)
S_1_	0.041929	0.513976	208.8924	97.35177	4.291497	162.78	98.1	18.22
S_2_	0.046729	0.515576	181.6807	97.35177	3.732457	158.61	98.9	14.2
S_3_	0.050505	0.516835	143.1054	97.35177	2.939966	154.45	99.35	11.21

Because of the high molar mass of the components involved, only a very small positive *v*_*2,s*_ value can be tolerated. The higher the value of *x*, weaker is the interaction between solvent and polymer, and stronger is the interaction among polymer chains. The polymer-solvent interaction parameter (*x*) has values that increased by increasing the content of crosslinker. For many systems, *x* was found to increase by increasing the polymer content for a given polymer volume fraction, smaller the value of *x*, greater the rate at which the free energy of the solution decreased by the solvent addition. As a result, liquids with the smallest *x* values are the best solvents for a polymer. When there is an increase in swelling ratio, *v*_*2,s*_ and *x* values are decreased. When there is a decrease in swelling ratio, the mesh size decreased leading to a decrease in *M*_C_ and the rate of diffusion of solute would also be expected to decrease [15, 28–34].

By increasing the content of EGDMA, the gel fraction increased while the sol fraction decreased. This can be credited to the development of intermolecular crosslinks. Table 5 is elaborating the effects of EGDMA contents on the gel fraction of AA–PVA hydrogel. As by increasing crosslinker concentration, there will be more crosslinking which will ultimately increase the gel fraction [4, 8, 35–37].

Due to the porous structure, hydrogels take in more water via capillary action and transfer the drug into the pores. Table 5 is elaborating the effects of crosslinking agent on porosity. By increasing the concentration of EGDMA, porosity decreased. As a result of increased amount of EGDMA, there was an increase in crosslinking density, decrease in hydrogel mesh size which resulted in decreased porosity [4, 8, 38–40].

### Drug release mechanism

When penetrant gets into the polymer network, the water soluble drug loaded in hydrogel is dissolved and drug diffusion occurs through the aqueous pathways to the surface of the device. The drug release was strongly linked to the swelling characteristics of the hydrogel which is a key parameter of structural design of the hydrogel. The method that best fits the release data was evaluated by the regression coefficient (r). Criterion for selecting the most appropriate model was based on the ideal fit indicated by the values of regression coefficient (r) near to 1.

Values of regression coefficient (r) for zero order, first order and higuchi models obtained from drug loaded AA–PVA hydrogels at varying content of EGDMA are given in Table 6. For the most of samples, the values of regression coefficient (r) obtained for first order release rate constants were found higher than those of zero order. It is attributed to the fact that drug release from the samples of varying degree of crosslinking are according to first order release. In higuchi model, r values at different crosslinker compositions indicated that the drug release mechanism is diffusion controlled [41].

**Table 6:** Effect of EGDMA concentration on release kinetics of AA–PVA hydrogel at different pH

Sample code	EGDMA content (% w/w)	pH	Zero order kinetics		First order kinetics		Higuchi Model	
			K_o_ (h¯^1^)	r	K_1_ (h¯^1^)	r	K_2_ (h¯^1^)	r
S_1_	0.082	1.2	2.221	0.976	0.026	0.983	0.104	0.995
		5.5	4.016	0.992	0.058	0.999	0.186	0.998
		7.5	5.833	0.976	0.137	0.997	0.274	0.996
S_2_	0.123	1.2	2.179	0.983	0.025	0.988	0.102	0.997
		5.5	3.953	0.99	0.056	0.997	0.183	0.998
		7.5	5.47	0.966	0.116	0.993	0.259	0.993
S_3_	0.165	1.2	2.028	0.98	0.023	0.984	0.094	0.994
		5.5	3.852	0.99	0.053	0.997	0.179	0.997
		7.5	5.407	0.963	0.108	0.988	0.256	0.99

Effect of EGDMA content on release exponent (n) is given in Table 7. The n value for the metformin HCl release was evaluated from the slope and intercept of the plot ln Mt/M_∞_ versus ln t and the results showed that the ‘n’ values are between 0.5 and 1.0 which indicated a non-fickian diffusion mechanism. It also clarified that the rate of drug diffusion from the hydrogels and the rate of polymer chain relaxation are interrelated [42].

**Table 7:** Effect of EGDMA concentration on release mechanism of AA–PVA hydrogel

Sample code	EGDMA content (%w/w)	pH	r	Release exponent (n)	Order of release
S_1_	0.082	1.2	0.983	0.991	non-fickian
		5.5	0.996	0.875	non-fickian
		7.5	0.995	0.665	non-fickian
S_2_	0.123	1.2	0.987	0.989	non-fickian
		5.5	0.994	0.929	non-fickian
		7.5	0.99	0.689	non-fickian
S_3_	0.165	1.2	0.989	0.999	non-fickian
		5.5	0.996	0.991	non-fickian
		7.5	0.985	0.741	non-fickian

### FTIR spectroscopy

**Fig. 4:**
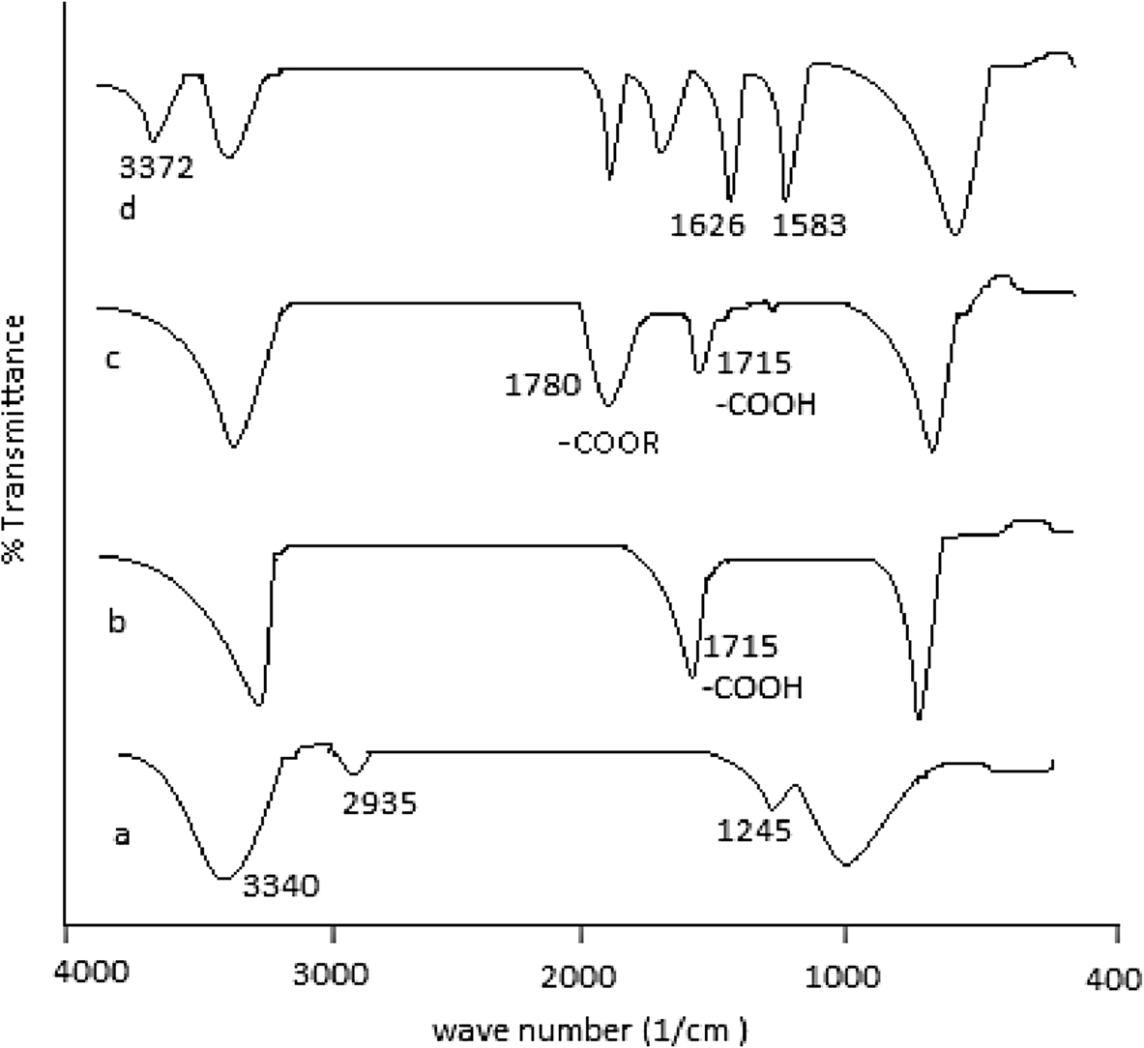
FTIR spectra of PVA (**a**), acrylic acid (**b**), AA–PVA unloaded hydrogel (**c**) and drug loaded AA–PVA hydrogel (**d**)

Comparative FTIR spectra of PVA, AA, AA–PVA hydrogel without drug and drug loaded AA–PVA hydrogel are shown in Fig. 4. The characterstic peak at 3340 cm^−1^ was due to the O–H stretching vibration of PVA and due to aliphatic C–H stretching vibration, a peak at 2935 cm^−1^ was observed. Due to C–O–C symmetrical stretching of the PVA backbone, a peak at 1245 cm^−1^ was seen. Peak at 1715 cm^−1^ was due to –COOH group of AA. A peak at 1780 cm^−1^ appeared for ester group in AA–PVA hydrogel representing the reaction between the –OH group of PVA with the –COOH group of AA [25]. In drug loaded AA–PVA hydrogel, a peak at 3372 cm^−1^ appeared representing N–H stretching of C = NH group of metformin and peaks at 1626 cm^−1^ and 1583 cm^−1^ appeared indicating C = N stretching of metformin [43].

### Scanning electron microscopy

**Fig. 5:**
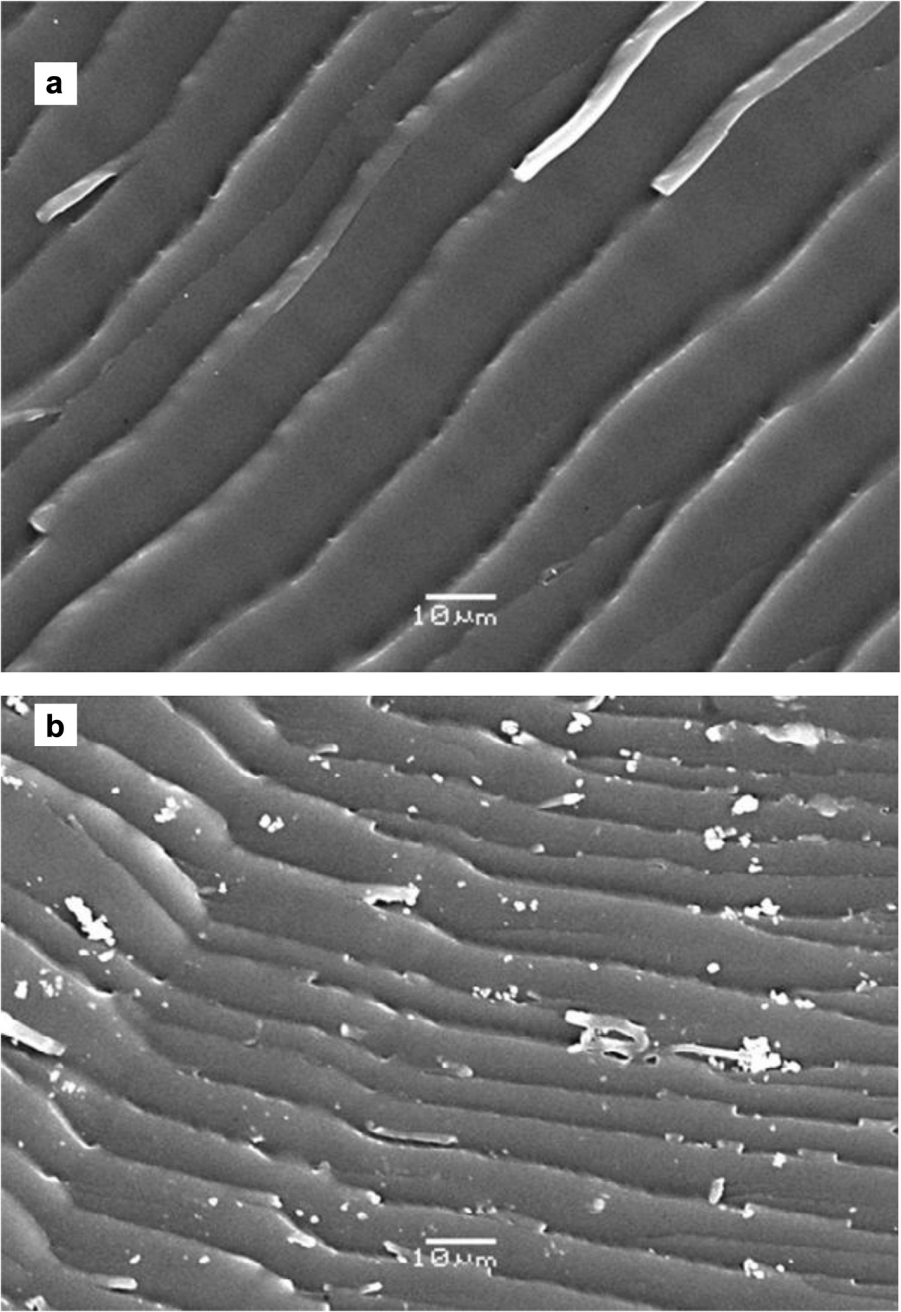
SEM images of S_1_ sample (**a**) and drug loaded S_1_ sample (**b**)

SEM images showed the voids present on the surface that will assist in drug incorporation. Fig. 5b is showing drug particles in hydrogel.

## Conclusion

Chemically crosslinked pH–sensitive AA–PVA hydrogels were synthesized in the presence of EGDMA&GA as crosslinkers and proved to be a good candidate for drug delivery to intestine. By increasing the content of EGDMA, a decrease in swelling and in drug release was noted due to more crosslinking. Gel fraction was found to increase by increasing the EGDMA concentration. Porosity was found to decrease by increasing the EGDMA content. Drug release followed first order and the mechanism was non-fickian diffusion in all cases. The FTIR confirmed the formation of graft polymer. SEM image of the drug loaded hydrogel showed incorporation of drug in the hydrogel along with the voids present on the hydrogel surface. S_1_–S_3_ samples can be effectively used as carriers for targeted drug delivery to intestine.
